# Regulatory Emotional Self-Efficacy Buffers the Effect of Heart Rate Variability on Functional Capacity in Older Adults With Chronic Low Back Pain

**DOI:** 10.3389/fpain.2022.818408

**Published:** 2022-05-20

**Authors:** Calia A. Morais, Lucas C. DeMonte, Emily J. Bartley

**Affiliations:** ^1^Division of Hematology and Oncology, University of Alabama at Birmingham, Birmingham, AL, United States; ^2^Department of Counseling and Higher Education, Northern Illinois University, DeKalb, IL, United States; ^3^Department of Community Dentistry and Behavioral Science, Pain Research and Intervention Center of Excellence, University of Florida, Gainesville, FL, United States

**Keywords:** emotional self-efficacy, heart rate variability (HRV), emotional regulation, older adult, low back pain

## Abstract

**Introduction:**

Chronic low back pain is one of the leading causes of disability globally among older adults. Prevailing research suggests that autonomic dysregulation places individuals at increased risk for chronic pain. This study examines the moderating role of emotional self-efficacy (ESE) on the relationship between heart rate variability (HRV) and pain related-outcomes, including movement-evoked pain (MEP) and physical functioning.

**Methods:**

In a secondary analysis of the Adaptability and Resilience in Aging Adults (ARIAA) study, a total of 58 adults (aged 60 and older) with chronic low back pain (cLBP) completed the PROMIS self-efficacy for managing emotions questionnaire and the 6-minute walk test (6 MWT) to assess functional capacity and MEP. Heart rate variability, indexed by the frequency domain, was assessed for 5 min during rest.

**Results:**

For pain-related outcomes, having a lower body mass index (*p* = 0.03) was associated with better functional capacity on the 6MWT, while higher education level (*p* = 0.01) and less pain duration (*p* = 0.00) were correlated with lower MEP. After controlling for sex, age, and body mass index, an increase in low-frequency HRV (LF-HRV) was associated with poorer physical functioning among individuals low in ESE (*b* = −0.12 *p* = 0.03). No significant moderation effects were observed for MEP.

**Conclusion:**

Our results bring attention to the degree to which ESE influences the relationship between LF-HRV and physical functioning. Interventions that enhance adaptive psychological processes such as ESE may dampen ANS dysregulation and mitigate risk for adverse pain outcomes among older adults with cLBP.

## Introduction

Chronic low back pain (cLBP) is a major musculoskeletal problem among older adults and the leading cause of years lived with disability (1–4). In fact, cLBP adversely impacts physical functioning (e.g., ability to exercise or perform daily functions) and quality of life and is one of the most therapeutically challenging pain conditions (5). Although the etiology of cLBP is likely multifactorial, evidence suggests that dysregulation in autonomic nervous system (ANS) function places individuals at increased risk for chronic pain (6). Specifically, the ANS plays a direct role in coordinating physical responsivity to stress (7). In the context of chronic pain, sustained or repeated arousal in physiological systems can contribute to allostatic load [i.e., a measure of physiological wear and tear on the body's regulatory systems (8)], thereby leading to maladaptive ANS function (i.e., increased sympathetic and/or decreased parasympathetic tone) (9). One of the potential consequences of allostatic load is chronic arousal of stress regulatory systems and subsequent development and persistence of chronic pain owing to sensitization of peripheral and central nervous system pathways. Likewise, a disruption in ANS balance is associated with deficits in cognitive and emotional regulation capacity, ultimately impairing adaptive responses to pain-related sensory and emotional stimuli (10, 11). Given the documented decrease in overall autonomic function and increase in chronic pain and pain sensitivity with age (12, 13), it is likely that pain and aging challenge the nervous system to adapt in the response of environmental stressors. Studies reporting altered ANS activity among older adults with cLBP are scarce, thus limiting understanding of the degree to which ANS dysregulation is responsible for the high prevalence of cLBP, which currently affects 21–75% of the older adult population worldwide (14).

The Model of Neurovisceral Integration (NMI) provides a theoretical framework highlighting the integration of physiological, emotional, and cognitive regulatory processes involved in stress adaptation (15, 16). This model posits that individuals with greater flexibility and adaptability to environmental demands have a greater capacity to regulate emotional and biobehavioral responses to stress, including pain. Autonomically-mediated heart rate variability (HRV), a metric reflecting the sequence of time intervals between successive heartbeats, has been proposed as a physiological resilience index of inhibitory control and adaptive regulation (17, 18). Evidence suggests that high resting HRV is indicative of autonomic flexibility and a highly adaptable nervous system including emotional self-regulation during stress (19, 20). Conversely, lower HRV serves as a biomarker for ANS dysfunction, as reflected by deficits in sympathetic and parasympathetic balance, thereby resulting in poor regulation of responses to emotional and pain stimuli (6, 21–23). Low HRV has been observed across various pain conditions and among individuals with lower functional capacity and higher perceived disability (24), suggesting that dysregulation of the ANS and reduced HRV may be implicated in the diathesis of chronic pain.

While HRV can be measured via time and frequency domains, frequency-based measurements (i.e., approach using spectral analysis to quantify the specific frequencies of beat-to-beat variation) have been recommended, as they concurrently assess sympathetic and parasympathetic activity (25). It is suggested that high frequency (HF) HRV is vagally-mediated and reflects parasympathetic activity, while low frequency (LF) HRV is a measure of baroreflex activity and hypothesized to consist of both sympathetic and parasympathetic influences (17, 26). Studies exploring these metrics at rest have found greater LF-HRV to be a marker of low pain unpleasantness and higher heat pain thresholds (17). Further, a 2014 systematic review found that experimentally induced pain dampens parasympathetic tone and engages baroreceptor activity, thereby resulting in decreased HF-HRV but higher LF-HRV (26). However, these studies were conducted in pain-free individuals, highlighting the need to improve understanding of the role of HRV in chronic pain populations, including older adults.

Despite the potential linkages among autonomic activity, emotional processes, and pain, surprisingly little is known regarding the role of emotion regulation (ER) on these relationships. ER is broadly characterized as a set of cognitive and attentional processes by which individuals modulate their emotional experiences to internal and external stimuli. Emerging research indicates that HRV shares neural networks with ER, with higher HRV associated with more regulated emotional responding (20). However, ER is not a unitary measure but rather is a multifaceted construct that influences interindividual differences in emotional responsivity. One such facet is regulatory emotional self-efficacy (ESE)—a construct entailing the subjective self-appraisal of one's capacity to modulate the intensity and expression of emotions (27). Evidence supports the role of ESE on a number of adaptive outcomes related to physical wellbeing (28) and mental health (29, 30), including less psychological distress among individuals living with chronic pain (31). Given that self-efficacy beliefs play a substantial role in self-regulative efforts associated with persistence in the face of difficulties, it is plausible that ESE may contribute to effective emotion regulation, thereby facilitating successful engagement in adaptive pain management efforts to improve pain and function. It is further postulated that dysregulation in ANS activity may serve as a risk factor for worse pain outcomes (10, 21), yet these effects may be mitigated by one's ability to successfully downregulate negative affective experiences. However, this is speculative and warrants further investigation.

The aim of this study was to examine the relationship between resting heart rate variability (i.e., LF-HRV, HF-HRV) with movement-evoked pain-MEP and functional capacity among older adults with cLBP, and explore the moderating role of ESE on these associations. As previously reported (17, 32, 33), we predict that HRV (LF and HF) will be associated with pain-related outcomes; however, to the best of our knowledge, no studies have yet explored these associations in a sample of older adults with cLBP.

## Materials and Methods

### Participants

This cross-sectional study was based on an analysis of the Adaptability and Resilience in Aging Adults (ARIAA) study that investigated the impact of resilience on pain modulatory capacity among older adults with cLBP (34–36). Sixty-nine adults (aged 60 years and older) with cLBP participated in the original study, and it was established that at least 60 participants would be necessary to achieve a power of 0.80 at *p* = 0.05 (two-tailed) for detecting associations of moderate to large effect sizes between measurements of psychological resilience and pain (37). Participants were included in the study if they self-reported at minimum mild low back pain (a score of 2 on a scale of 10 ranging from 0 “no pain” to 10 “most intense pain imaginable”) occurring on at least half of the days during the past 3 months. On average, participants reported a pain level of 5.4/10 (SD = 1.7). Enrollment in the study was not solely restricted to cLBP given the high incidence of other medical comorbidities experienced by aging adults; however, low back pain had to be identified as the primary pain condition. Participants were excluded for the following: recent vertebral fracture; back surgery within the past 6 months; diagnosis of cauda equina syndrome; uncontrolled hypertension (≥150/90); current cardiovascular disease; neurological disease associated with somatosensory abnormalities (e.g., neuropathy, seizures, Parkinson's disease); current major medical illness (e.g., metastatic or visceral disease); chronic opioid use; and systemic inflammatory disease (e.g., spondyloarthropathies including ankylosing spondylitis, systemic lupus erythematosus, etc.).

### Procedures

The University of Florida Institutional Review Board approved all study procedures. Participants were recruited from the community through posted fliers, radio and print media announcements, word-of-mouth, and physician referrals. Following an initial phone screen to determine study inclusion, eligible participants attended two 2.5-h visits scheduled approximately 1 week apart from one another. During the first visit, participants provided written and verbal consent. Participants completed a demographic and medical history assessment, anthropometric tests measuring body composition, psychosocial questionnaires, and the 6 Minute Walk Test (6 MWT). Participants' resting heart rates were also calculated prior to beginning the 6 MWT and testing was discontinued if the resting heart rate was >120 or <50 bpm. Additional questionnaires were sent to participants to be completed at home in-between visits 1 and 2. During the second visit, additional questionnaires to assess psychosocial functioning (e.g., pain catastrophizing) were administered (34–36), and HRV was measured. Prior to HRV testing, participants were provided with a 30-min rest period whereby health status was reviewed, study questionnaires were completed, and three consecutive measurements of blood pressure were obtained. Participants were provided up to $100 compensation for completion of the study.

### Measures

#### Heart Rate Variability

Participants were asked to not eat or drink anything except water or exercise for 4 h prior to the study visit. In addition, they were asked to abstain from nicotine use for 2 h and alcohol use for 12 h prior to the session. Consistent with existing procedures and previous studies conducted by our group (22, 38), heart rate variability (HRV) was assessed with a three-lead BioCom model 3000 Heart Rhythm Scanner (Biocom, Technologies) with the electrical leads positioned on the left 2nd rib (ground), right 2nd rib, and lower left torso (rib cage boundary below the left breast) to acquire and process heart rate data. Electrocardiography (ECG) signals were recorded and checked for impedance (below 300 KΩ), digitized (1,024 samples per second), and visually inspected for errors using the Biocom Heart Rhythm Scanner software (version 2.0). Participants were not exposed to the visual displays. As commonly described in previous pain studies (22), HRV was measured for 5-min at rest, while participants were placed in a recliner (supine position) for the testing. Participants were instructed to rest quietly but not fall asleep. All data collection occurred in the mornings within a 2-h time window (i.e., 8–10 a.m.) and at the same physical location (laboratory space at the University of Florida).

HRV frequency analysis was specifically utilized in this study for a more nuanced separation between sympathetic and parasympathetic irregularities. Measurement and interpretation of HRV were based on recommendations provided by the Task Force of the European Society of Cardiology and the North American Society of Pacing and Electrophysiology (39). Per recommendations, the absolute power distribution of heart rate oscillations can be divided into the low-frequency (LF) band (0.04–0.15 Hz) and high-frequency (HF) band (between 0.15 and 0.4 Hz) (39). The range in Hz (Hertz) refers to the specific location or peak of the band of frequency, whereas the total power (TP) reflects overall autonomic activity between the sympathetic and parasympathetic branches and is calculated using a frequency range from 0 to 0.4 Hz (25). The TP for frequency domain measures are calculated in milliseconds squared (ms^2^) and had the following limits based upon prior research: Total Power 10–10,000 ms^2^, Low Frequency (LF) 10–6,000 ms^2^, and High Frequency (HF) 10–6,000 ms^2^ (40).

#### PROMIS Self-Efficacy for Managing Emotions-Short Form 8a

The PROMIS Self-Efficacy for Managing Emotions measures one's confidence to manage emotions such as anxiety, helplessness, and discouragement (41). The questionnaire includes 8 items using a scale from 1 (“I am not at all confident”) to 5 (“I am very confident”), with a score range of 8–40. Participants are asked to rate their “current” level of confidence to statements such as “I can handle negative feelings” or “I can relax my body to reduce my anxiety.” Higher scores indicate greater levels of ESE. The scale has a high internal consistency range from 0.90 to 0.95 for the 8-item short forms (41). In our sample, the internal consistency was also high (α = 0.95).

#### Six-Minute Walk Test

The 6 MWT is a commonly used performance-based measure used in exercise rehabilitation and clinical research to evaluate physical capacity and cardiovascular function in older adults (42). Participants walk back and forth on a 20-meter (65 ft) segment of a straight, flat, unimpeded hallway for a period of 6 min. During the assessment, participants were instructed to walk as far as possible, but not to run or jog. A lower score reflects less distance covered in 6 min, suggesting worse physical functioning and greater disability. Given that measures of dynamic pain during activity have been suggested to be a stronger predictor of pain-related disability than spontaneous pain measures (43–45), movement-evoked pain (MEP) was assessed by having participants rate their overall lower back pain experienced during the 6 MWT. Ratings were made on a numerical rating scale ranging from 0 “no pain” to 100 “most intense pain imaginable” and were obtained immediately following the completion of the 6 MWT. The 6 MWT is a safe, low-cost, valid, and reliable test (α = 0.91) (42, 46). Our method of measuring MEP is consistent with existing approaches (47), as well as other studies by our research group (48).

### Statistical Analysis

All analyses were conducted using SPSS 27.0, and the significance level was set at *p* ≤ 0.05 (two-tailed). Means, standard deviations, and counts for demographic characteristics were calculated using descriptive statistics. Pearson's correlations were performed to examine the relationship between demographic characteristics and study variables. Regression analyses were conducted to evaluate the moderating effect of ESE on the relationship between HRV (LF-HRV; HF-HRV) and pain and functional capacity outcomes (i.e., MEP and 6 MWT). We controlled for sex and age, due to the impact of these variables on HRV (49) and included body mass index (BMI) as a covariate due to its association with increased risk for worse pain-related outcomes (50, 51). In these analyses, sex was dummy coded as follows: 0 = female and 1 = male. The conditional effects of HRV were tested at three levels of ESE: one standard deviation below the mean, at the mean, and one standard deviation above the mean. Analyses were conducted using PROCESS (52), a tool that uses regression-based path-analytic modeling and automatically produces mean centering and conditional effects for moderation models. Cohen's *f*^2^ (small = 0.02, medium = 0.15, large = 0.35) was used to calculate effect sizes associated with significant findings from linear regression analyses (53).

## Results

### Sample Characteristics

The original sample size was 69; however, a total of nine participants were excluded from the analyses due to no longer meeting eligibility during in-person study sessions (*n* = 1 use of exclusion medication, *n* = 3 not meeting pain duration criteria, *n* = 3 exclusionary medical condition, *n* = 2 having time constraints to continue study participation). In addition, HRV data were not collected on two participants due to a malfunction in the HRV software, leaving 58 participants for the analysis reported in this article. Demographic characteristics are reported in Table 1. As seen, most participants were female (57.9%) and the average age was 68 years (SD = 7.2). The duration of low back pain was 15.9 years (SD = 13.5). On average, the total distance covered during the 6 MWT was 390.6 meters (SD = 83.5), and average MEP was 26.9/100 (SD = 27.3). Mean HRV values are as follows: LF-HRV = 269.7 ms^2^, SD = 565.1; HF-HRV = 275.8 ms^2^, SD = 883.5. These values are consistent with previous research conducted with pain samples (40). See Supplementary Table 1 for additional information regarding physiological measures.

**Table 1:** Demographic and clinical characteristics.

**Characteristic**	**M or N**	**SD or %**
**Age (years)**	68.2	7.1
**Sex**		
Female	33	56.9
Male	25	43.1
**Race**		
Black/African American/Other^**^	17	29.3
White/Caucasian	41	70.7
**Ethnicity**		
Hispanic	3	5.2
Not hispanic	55	94.8
**Marital status**		
Married/partnered	29	50.0
Not married/partnered	29	50.0
**Employment**		
Employed	8	13.8
Not employed/retired	50	86.2
**Education**		
<High school diploma	12	20.7
Some college/technical school	17	29.3
Associates/bachelors	17	29.3
Graduate/professional	12	20.7
Annual Income^*^		
<$20,000	20	34.5
$20,000–39,999	10	17.2
$40,000–59,999	11	19.0
$60,000–99,999	7	12.1
≥$100,000	7	12.1
BMI (kg/m^2^)	28.9	5.2
Back pain duration (years)	15.9	13.5
6 MWT-MEP (0–100)	26.9	27.3
6 MWT-Distance (meters)	390.6	83.4
ESE (8–40)	29.4	6.7

* *Some data not reported. BMI, body mass index; 6 MWT, 6-minute Walk Test; MEP, movement-evoked pain; ESE, emotional self-efficacy*.

** *Other includes Asian, Hawaiann and some other race*.

### Bivariate Correlations

Table 2 presents the bivariate correlations for the associations between participant characteristics and study variables. Being older and married was associated with greater ESE (*p* = 0.01 and *p* = 0.02, respectively). Further, there was a positive correlation between HF-HRV and age (*p* = 0.04). Regarding pain and functional capacity outcomes, having a lower BMI (*p* = 0.03) was associated with increased walk capacity. In addition, having less education (*p* = 0.01) and a greater pain duration (*p* = 0.00) was associated with greater MEP. Finally, greater 6 MWT distance was associated with less MEP (*p* = 0.00). There were no additional significant correlations in the analysis (*ps* > 0.05), including a lack of association between HRV metrics with MEP and functional capacity.

**Table 2:** Bivariate correlation matrix.

**Variable**	**1**	**2**	**3**	**4**	**5**	**6**	**7**	**8**	**9**	**10**	**11**	**12**	**13**	**14**
1. Age	1.00													
2. Sex	0.20	1.00												
3. Race	−0.27^*^	0.13	1.00											
4. Education	0.01	−0.16	−0.42^**^	1.00										
5. Marital status	−0.31^*^	−0.10	0.34^**^	−0.17	1.00									
6. Employment	0.28^*^	0.15	0.15^*^	−0.08	−0.10	1.00								
7. Income	0.35^**^	−0.05	−0.31^*^	0.24	−0.44^**^	−0.21	1.00							
8. BMI	−0.07	−0.04	0.24	−0.12	0.23	−0.02	−0.22	1.00						
9. Pain duration	0.07	0.05	−0.15	0.05	−0.10	0.11	0.07	−0.16	1.00					
10. LF-HRV	0.24	0.18	−0.04	0.07	0.10	−0.03	0.11	−0.02	−0.09	1.00				
11. HF-HRV	0.26^*^	0.15	−0.00	0.02	0.13	−0.06	0.07	−0.06	−0.04	0.80^**^	1.00			
12. ESE	0.33^*^	0.15	−0.08	0.09	−0.30^*^	0.02	0.25	0.02	0.11	0.14	0.09	1.00		
13.6MWT-function	−0.23	−0.13	−0.26	0.26	−0.09	−0.18	0.16	−0.29^*^	0.17	−0.04	0.02	0.07	1.00	
14.6MWT-MEP	0.14	0.22	0.12	−0.35^**^	0.13	0.24	−0.26	0.19	−0.41^**^	0.05	0.02	−0.15	−0.57^**^	1.00

*Race coded: 0, white 1, black or other; sex coded: 0, female, 1, male; marital status coded: 0, married, 1, not married; education coded: 0, ≤high school degree, 1, >high school degree; employment status: 0, employed, 1, not employed; income coded: 0, <$20,000, 1, ≥20,000; LF-HRV, low-frequency heart rate variability; HF-HRV, high frequency heart rate variability; ESE, emotional self-efficacy; 6MWT, six-minute walk test; MEP, movement-evoked pain*.

* *p < 0.05*,

** *p <0.01*.

### Moderation Analyses

#### Six-Minute Walk Test: Functional Capacity

**Figure 1:**
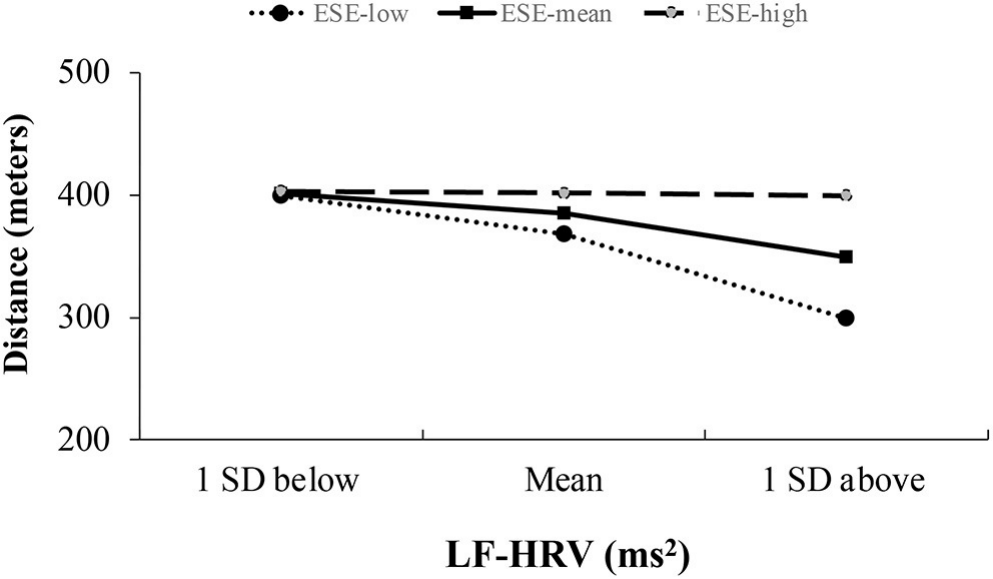
Association between LF-HRV and 6 MWT distance across low, mean, and high levels of emotional self-efficacy (ESE).

Table 3 presents the results for the effect of ESE on the relationship between LF-HRV, HF-HRV, and functional capacity, after controlling for sex, age, and BMI. The overall model for LF-HRV accounted for 27.7% of the variance observed in functional capacity (*F* = 3.19, *R*^2^ = 0.27, *p* = 0.01). There was a significant interaction between LF-HRV and ESE [*b* = 0.01, Δ*R*^2^ = 0.09, *F* = 6.09, *p* = 0.02, (Cohen's *f*^2^ = 0.16, medium effect)]. As seen in Figure 1, as LF-HRV increased (1 SD above mean), there was less distance covered in the 6 MWT (i.e., poorer functional capacity) among individuals with a lower level of ESE (*b* = −0.12 *p* = 0.03). These effects were not significant for average (*b* = −0.06 *p* = 0.06) or high (*b* = −0.00 *p* = 0.84) ESE. The overall moderation model for HF-HRV, after controlling for covariates, did not explain a significant amount of variance in functional capacity (*F* = 2.02, *R*^2^ = 0.19, *p* = 0.08) and there were no significant interaction effects observed between HF-HRV and ESE (*p* = 0.88).

**Table 3:** Moderation analysis for HRV and pain-related outcomes.

**Variable**	**6 MWT-function**			**6 MWT-pain**		
	***b***	***SE***	***p***	***b***	***SE***	***p***
**(A)**						
Age	−4.19	1.56	0.01	0.82	0.55	0.14
Sex	−9.81	21.29	0.65	11.95	7.50	0.12
BMI	−5.38	1.91	0.01	1.10	0.67	0.11
LF-HRV	−0.06	0.03	0.06	0.01	0.01	0.56
ESE	2.57	1.64	0.12	−1.10	0.58	0.06
LF-HRV X ESE	0.01	0.00	0.02	−0.00	0.00	0.50
**(B)**						
Age	−3.62	1.66	0.03	0.72	0.55	0.20
Sex	−21.36	22.01	0.34	12.95	7.38	0.09
BMI	−5.06	2.02	0.02	1.05	0.68	0.13
HF-HRV	0.01	0.02	0.73	−0.00	0.00	0.64
ESE	2.38	1.74	0.18	−1.11	0.58	0.06
HF-HRV X ESE	0.00	0.00	0.88	0.00	0.00	0.65

*Sex coded: 0, female, 1, male; HF-HRV, high frequency heart rate variability; LF-HRV, low frequency heart rate variability; ESE, emotional self-efficacy; 6 MWT, six-minute walk test*.

#### Six-Minute Walk Test: Movement-Evoked Pain

In the examination of ESE as a moderator of the HRV and MEP relation, the overall model was not significant for LF-HRV (*F* = 1.64, *R*^2^ = 0.17, *p* = 0.16) or HF-HRV (*F* = 1.60, *R*^2^ = 0.16, *p* = 0.17). There were also no significant interaction effects detected for LF-HRV (*p* = 0.50) or HF-HRV (*p* = 0.65) and ESE with MEP.

## Discussion

While the pathophysiology of chronic pain is multifactorial, ANS dysregulation (as indexed by HRV) has been touted as a potential contributor. HRV reflects the ability to generate regulated emotional and arousal responses through the ANS and serves as an index of physiological resilience and emotion regulatory capacity. Greater HRV, as reflected by enhanced parasympathetic tone, signifies adaptive autonomic flexibility and emotional regulation in the context of stress (e.g., pain), while lower HRV is associated with greater sympathetic activation and adverse health-related function including psychological rigidity (16, 19). Although a number of studies, including recent meta-analyses, have observed reduced HRV in individuals with chronic pain, including higher mean LF-HRV and lower HF-HRV among patients with low back pain (21), the extent to which psychological factors contribute to differences in the association between autonomic regulation and pain has been relatively unexplored (21). Therefore, the aim of this study was to assess ESE as a moderator between HRV and pain and functional capacity outcomes (MEP, physical function) among older adults with cLBP.

Overall, the results of this study support the Model of Neurovisceral Integration by suggesting an interplay between physiological and emotional regulation processes in the context of pain-related adaptation. Specifically, we found that higher resting LF-HRV was associated with poorer functional capacity in adults with lower ESE, but not among those higher in ESE. While these findings are in contrast to a previous study finding higher LF-HRV to be associated with reduced ratings of unpleasantness and higher thresholds to experimental pain (17), it is possible that inconsistency in results might be in part due to differences in study samples (older vs. younger cohorts, chronic pain vs. pain-free individuals), which could impact how ANS activity affects pain and function.

It is also important to note that cognitive and emotional appraisals, such as self-efficacy, might play an important role in modulating autonomic responses, an effect which may alter functional capacity. Specifically, individuals with a lower capacity for regulating negative emotions may have alterations in sympathetic baroreflex engagement; in turn, this may potentiate negative downstream effects on pain-related function. Although there were no significant effects observed for MEP, findings suggest that the capacity for and confidence in emotional regulation may have a greater impact on one's motivation and ability to engage in performance-related tasks, as compared to self-evaluations of pain.

Numerous studies have linked self-efficacy with more adaptive functioning in chronic pain including lower pain intensity and disability, greater health-related quality of life, and higher functional capacity (54–56), Similarly, Agar-Wilson and Jackson found that efficacy in emotion regulation was associated with higher quality of life and reduced negative affect in people with chronic pain (57). While speculative, limited emotional awareness and lower confidence in affect regulation may partially explain why some individuals experience difficulty engaging in adaptive pain management efforts, especially in relation to activities that may be emotionally and physically challenging and/or exacerbate pain (e.g., exercise). While this warrants further investigation, findings from the current study have potential clinical relevance. In particular, therapeutic approaches such as acceptance and commitment therapy and mindfulness-based stress reduction focus on increasing awareness, tolerance, and non-reactivity of negative emotions. Coupled with treatments that improve HRV (e.g., biofeedback, mindfulness meditation) (58, 59), targeting ESE through strategies such as these may promote adaptive emotion regulation during times of stress (e.g., pain flare-ups) and subsequently dampen dysregulation in physiological regulatory systems that increase risk for adverse pain outcomes.

Interestingly, results varied across HRV levels with the moderation effect of ESE on functional capacity only observed for the low frequency band. While HF-HRV is largely driven by vagally-mediated parasympathetic activation, the clinical significance of LF-HRV is more complex although studies have proposed it as a measure of baroreflex activity, thereby having both sympathetic and parasympathetic influences (17, 26). Though limited, previous research has reported an inverse relationship between LF-HRV and experimentally-induced pain (17), Although our findings suggest that functional capacity may be particularly impacted by baroreflex activity, future studies are encouraged to examine how the interactive effects of psychological processes on HRV impact pain-related outcomes.

## Strengths and Limitations

To our knowledge, this is the first investigation exploring the role of ESE on the relationship between HRV and pain outcomes among older adults. We included participants who reported, on average, a moderate level of pain intensity during the screening process. Given that a moderate level of pain is often associated with greater functional limitations (60, 61), findings from our study provide support for examining emotional and physiological factors that may dampen the negative impact of pain on functioning. Obtaining an understanding of the self-beliefs associated with emotion regulation offers an opportunity to target views and attitudes that could enhance the capacity to manage emotions more effectively in response to pain. Further, MEP is thought to be the primary driver of poor mobility and functioning, particularly among older adults; (43) therefore, the examination of functional capacity and MEP provides unique information regarding mobility limitations and activity-induced pain associated with cLBP.

These findings should be considered in light of their limitations. We did not measure baseline pain prior to obtaining MEP following the 6 MWT task; therefore, it is unclear if some participants might have experienced pain relief from the physical task instead of increased pain. Additionally, while the 6 MWT is a widely used measure of functional capacity, it is possible that other measures (e.g., sit-to-stand, ascending/descending stairs) may be of greater relevance to disability and functional capacity in cLBP. As this study was conducted in older adults with cLBP, the current findings may not generalize to other demographic groups or pain conditions. It is important to point out that we examined self-efficacy beliefs of emotion regulation and more research is needed to examine the utility of this assessment in clinical settings. Furthermore, we did not measure the act of employing coping skills to self-regulate, thus our findings may not reflect an individual's true level of self-regulation and use of specific coping skills. Further, establishing the association between HRV and cLBP among older adults remains obscured due to the factors that influence or partially increase risk for adverse pain outcomes as well as changes in ANS functioning over time. This is compounded by biopsychosocial mechanisms that vary across aging populations, as trauma, mental illness, discrimination, and hardship, among others serve as important contributors to health behaviors, as well as the prevalence and experience of chronic pain (62, 63). While our study included minoritized groups (i.e., African Americans, Hispanics) and diverse levels of education and income, future studies should also aim to examine the role of patient characteristics and the intersectionality of identities to further understand how patient characteristics may influence the impact of these findings.

Furthermore, the emotional states and stress associated with these lived experiences affect ANS function and subsequently influence one's ability to respond to stress, including pain. It is also important to note that we assessed HRV from a single recording; therefore, future studies using repeated measurements over time are warranted to confirm and replicate results. Lastly, while the study was adequately powered and we observed a significant interaction of medium effect, future studies would benefit from a larger sample size to confirm the study results and continue to clarify the role of low-frequency measures of HRV in pain and affective processing.

## Conclusion and Future Directions

The sympathetic and parasympathetic nervous systems play an important role in pain regulation; however, there has been limited investigation of factors that influence the association between autonomic function and pain outcomes. Overall, we found a significant moderating effect of ESE on the association between LF-HRV and functional capacity, suggesting that individuals with lower emotional regulatory efficacy and higher LF-HRV may be more susceptible to impaired functional capacity. Future studies should be conducted with a larger sample size to confirm the study results and continue to clarify the role of low-frequency measures of HRV in pain and affective processing. Further, deepening our understanding of these relationships may inform intervention strategies to increase patients' confidence in their ability to employ emotion regulation skills in response to pain while simultaneously mitigating pain-related interference.

## Data Availability Statement

The raw data supporting the conclusions of this article will be made available by the authors, without undue reservation.

## Ethics Statement

The studies involving human participants were reviewed and approved by Institutional Review Board-University of Florida. The patients/participants provided their written informed consent to participate in this study.

## Author Contributions

EB contributed to study conceptualization and study design. CM conducted the statistical analysis. All authors contributed to the write-up and revision of the manuscript. All authors contributed to the article and approved the submitted version.

## Funding

The research reported in this publication was supported by NIH/NIA Grant (K99AG052642 and R00AG052642) awarded to EB and NIH/NIA Grant (5P30AG059297-04S1) provided to the University of Florida (CM).

## Conflict of Interest

The authors declare that the research was conducted in the absence of any commercial or financial relationships that could be construed as a potential conflict of interest.

## Publisher's Note

All claims expressed in this article are solely those of the authors and do not necessarily represent those of their affiliated organizations, or those of the publisher, the editors and the reviewers. Any product that may be evaluated in this article, or claim that may be made by its manufacturer, is not guaranteed or endorsed by the publisher.
