# Supplementary material for: Regulatory Emotional Self-Efficacy Buffers the Effect of Heart Rate Variability on Functional Capacity in Older Adults With Chronic Low Back Pain
Source: Front Pain Res (Lausanne). 2022 May 20;3:818408. doi: 10.3389/fpain.2022.818408 (PMC9163301; doi:10.3389/fpain.2022.818408)
Supplement: Supplementary file 1 [file Table_1.pdf]

Supplementary Table 1. ***Descriptives of Physiological Measurements***

**Parameter**	M	SD
Systolic Blood Pressure (mmHg)	127.3	15.0
Diastolic Blood Pressure (mmHg)	71.3	8.7
HR (bpm)[a]	63.9	9.2
Total Power (ms^2)	898.1	2006.9
Very Low Frequency(ms^2)	269.7	565.1
HFn (n.u.)[b]	41.9	22.5
LFn (n.u.)[c]	58.1	22.5
LF/HF Ratio (ms^2)	2.9	4.1
SDNN (ms)[d]	47.3	39.7
RMSSD (ms)[e]	41.6	58.7

a= Heart rate; b=High frequency power in relation to total power; c=Low frequency power in relation to total power
d=Standard deviation of consecutive RR intervals; e=Root mean square of the consecutive RR differences
